# Psychotherapeutic Competencies in Blended Care Psychotherapy: Development of a Competence Framework

**DOI:** 10.2196/100125

**Published:** 2026-09-16

**Authors:** Gerald Marc Weiher, Mirjam Naomi Woide, Maria Zirenko, Michael Tremmel, Ulrich Stangier, Holger Horz

**Affiliations:** 1 Department of Educational Psychology, Institute of Psychology, Goethe University Frankfurt, Theodor-W.-Adorno-Platz 6, Frankfurt am Main, Hesse, 60629, Germany, 49 69 86004468; 2 Department of Clinical Psychology, Institute of Psychology, Goethe University Frankfurt, Frankfurt am Main, Hesse, Germany

**Keywords:** blended care psychotherapy, psychotherapeutic competencies, competence framework, digital competencies, digital psychotherapeutic interventions

## Abstract

**Background:**

Blended care psychotherapy (BCP) combines face-to-face psychotherapy with digital interventions, such as videoconferencing, apps, or virtual reality, which can be incorporated in various ways and at different stages of the therapeutic process. Despite its increasing implementation, conceptual and terminological inconsistencies continue to impede a shared understanding of the competencies required for its effective delivery.

**Objective:**

This theoretical paper introduces a framework of psychotherapeutic competencies in BCP, designed to provide conceptual clarity.

**Methods:**

The framework was developed in four steps. (1) An exploratory literature search was conducted to identify existing competence and educational frameworks relevant to psychotherapy, digitalization, and educational psychology. (2) These models informed a preliminary theoretical framework. (3) This preliminary framework was critically reviewed and refined in an expert workshop with 5 licensed psychotherapists and researchers experienced in BCP. (4) Lastly, findings from a systematic review of 35 studies on BCP competencies were integrated, resulting in the final 8-domain framework.

**Results:**

The resulting framework comprises *Modalities* of BCP (eg, face-to-face, videoconferencing, and apps), *Competence Facets* (knowledge, skills, and attitudes), as well as 8 *Competence Domains* (Creation of Setting and Infrastructure; Transfer of Communication and Methods; Therapeutic Relationship; Self-Regulation, Self-Confidence, and Self-Care; Data Security, Legal Issues, Ethics, and Safety; Technological Aspects; Scientific Knowledge and Methods; and Individual and Cultural Diversity).

**Conclusions:**

This conceptual contribution aims to strengthen the theoretical foundation of BCP, support competence-based education for psychotherapists, and guide future research toward the empirical validation and refinement of BCP-specific competencies.

## Introduction

### Digital Integration and Benefits in Psychotherapy

The integration of digital elements into psychotherapy is commonly referred to as blended care psychotherapy (BCP), which denotes a combination of traditional face-to-face treatment with digital modalities [1-5]. However, psychotherapists can integrate BCP in different ways, ranging from the delivery of psychotherapy via digital communication technologies (eg, telephone or videoconferencing) to the use of additional digital adjuncts, such as apps, websites, text messaging, virtual reality, or other technology-based tools that complement psychotherapeutic treatment. Accordingly, digital formats vary in the requirements they impose on practitioners and in the degree to which therapists are involved in their implementation [6]. Meta-analytic findings indicate that technology-assisted interventions and traditional face-to-face psychotherapy result in comparable outcomes [7-9] or show medium (therapy via a web application with clinical support) to large (therapy via video or chat) effect sizes compared with a waiting list [10]. Furthermore, technological applications can improve access to psychotherapeutic care, for example, for people in rural areas or those with limited mobility. At the same time, they enable psychotherapeutic interventions to be conducted regardless of location and, in some cases, with flexible scheduling [11]. Additionally, the integration of technological applications into psychotherapy can facilitate psychotherapeutic processes, for example, through the automated transcription and documentation of psychotherapy sessions [12]. Nevertheless, the implementation of digital tools and interventions comes with the necessity of responsible use of these systems [13-15]. Moreover, in line with the Cognitive Load Theory [16], BCP settings may increase cognitive load, as an additional component must be managed, thereby increasing the demands placed on psychotherapists [16,17]. Compared to face-to-face psychotherapy, psychotherapists report that BCP is more tiring and strenuous [18-20]. Therefore, the digital environment and new digital tools within psychotherapy challenge the sufficiency of currently defined psychotherapeutic competence domains. Furthermore, they address the necessity of definition and conceptualization of psychotherapeutic competencies in BCP that are crucial for the ethical, reasonable, and responsible use of digital technology [13,21]. This poses the question of how to define the construct competencies of psychotherapists working with BCP and to define the necessary competence domains. Furthermore, the necessary competence raises the question of how they could be measured and developed, given existing barriers to the acceptance of technology in psychotherapy. The aim of the following conceptual paper is to present the first step in developing a framework on competencies in BCP.

### What Is Competence?

There exist numerous definitions of the term “competence” and ways to conceptualize it within the psychotherapeutic field [22-27]. We follow the understanding that the term “competence” describes the personal prerequisites needed to successfully cope with specific situations [28]. Furthermore, competence is understood as including knowledge, skills, and attitudes necessary for the performance of professional activities. This conceptualization is consistent with competence-based approaches in professional psychology, which view professional competence as a multidimensional construct encompassing not only knowledge and skills but also attitudes [22,23,25-27,29-31]. Recent work further emphasizes that attitudes constitute an integral component of competent psychological practice rather than merely representing contextual factors or predictors of behavior [32].

Accordingly, knowledge is described as the information base for competencies and activities and, therefore, the mental representation of procedures and structures [26]. Knowledge also encompasses procedural understanding, that is, knowing how to perform or create something. In the context of clinical psychology, this, for example, involves a deeper understanding of how to conceptualize and achieve specific goals, such as possessing the knowledge required to guide a patient through relaxation techniques aimed at reducing anxiety [33]. Skills are described as specific cognitive or motor abilities that are typically developed through a process of training and practice [26]. Skills encompass the ability to apply knowledge effectively, which in the context of psychotherapy is demonstrated, for example, by the ability to write a case conceptualization that uses the appropriate concepts to address a specific problem [23,34]. Attitudes include feelings, values, and beliefs that influence a person’s behavior and performance [26]. For psychotherapists, attitudes have been shown to play an important role in the adoption of BCP [35].

### What Is Psychotherapeutic Competence in the Field of BCP?

The investigation of competencies, as well as specific competencies in a digital setting, is relatively new in the field of psychotherapy [36], though there is increased scientific interest in digital competence [37]. However, the field of educational psychology offers a long history of engagement with this field of competencies and digital competencies, as well as their development [38]. Different models exist, and various definitions of digital competencies are provided [39,40]. In line with Ferrari [40], who provided a report on the European project Digital Competence, digital competence is defined as a “set of knowledge, skills, and attitudes (thus including abilities, strategies, values, and awareness) that are required when using […] digital media to perform tasks; solve problems; communicate; manage information; collaborate; create and share content; and build knowledge” (p. 3). In the domain of psychotherapy, it is of the utmost importance that practitioners possess the requisite competencies for delivering BCP in an effective and ethical manner [13,21,41]. It has been postulated that improvement in psychological treatments, in particular digital mental health and blended care interventions, will be achieved by defining competence profiles for clinical training [13,15,21,42]. In accordance with the competence definitions provided above, in the context of BCP we propose the following definition: “psychotherapeutic competencies for BCP describe the knowledge, skills, and attitudes necessary for the successful, evidence-based delivery of psychotherapy in conjunction with digital media” [43] (p. 2).

### Existing Frameworks for Digital Competencies for Psychotherapists

There are certain domains of digital competence that have been identified as relevant to the field of psychotherapy. Frameworks and reviews describe lists of certain behaviors, skills, ethical necessities, and knowledge aspects that psychotherapists need to develop [15,21,25,44-47]. For example, Maheau et al [25] provide a list of more than 51 telebehavioral objectives and 149 individual telebehavioral practices within 7 domains. The models show insights into important aspects of digital competence within the field. However, these models also show certain difficulties. On a theoretical level, they do not discuss and are not based on already existing theoretical contributions in the field of digital competence, such as the Technological Pedagogical Content Knowledge (TPACK) framework [48] or the unified theory of acceptance and use of technology (UTAUT) model [49]. This hinders integration with existing research and the insights derived from it. In line with this argumentation, competence domains are hardly being grasped, and it is difficult to develop training based on the existing lists. Moreover, existing diagnostic tools for psychotherapists’ digital competencies measure competence in a generalized manner [50]. Furthermore, and probably most importantly, the existing approaches are not specifically conceptualized for BCP [15,21,25,44], which makes it difficult to transfer these frameworks to that setting.

### Aims

Accordingly, the aim of the present study was to develop a theoretically grounded framework that integrates relevant perspectives on psychotherapeutic competencies in BCP. Specifically, the framework was designed to (1) conceptualize competencies required for BCP, (2) integrate different BCP *modalities*, *competence facets* (knowledge, skills, and attitudes), and *competence domains* within a coherent framework, (3) enhance practical applicability by offering a structured approach that enables practitioners, researchers, and educators to identify and address competence requirements, and (4) provide a foundation for future competence assessment and training development. The contribution of this framework is not the introduction of entirely new psychotherapeutic competencies; rather, its contribution lies in specifying how established psychotherapeutic competencies are reorganized and contextualized for BCP. In particular, the framework makes explicit how different digital modalities require different configurations of knowledge, skills, and attitudes across competence domains.

## Methods

### Development of the Competence Framework

To address the outlined issues, we have developed a theoretically based competence framework built on existing competence models. The initial framework was refined during an expert workshop and afterward complemented by the results of a systematic review [43] to establish the final framework that was not part of the expert workshop. The process is described below.

### Building on Existing Models: Educational and Clinical Psychology Competence Models

#### Theoretical Frameworks for Competence Development

An exploratory literature search was conducted to identify influential theoretical models relevant to competence development, psychotherapeutic competencies, technology integration, and telepsychology competencies. The following models were selected as theoretical reference frameworks because they are widely established and frequently cited within the respective fields of educational psychology, professional psychology, technology acceptance, and telepsychology. They represent influential frameworks that could inform different components of psychotherapeutic competencies in BCP.

#### TPACK Model

The TPACK model was developed as a framework for the complex teacher knowledge necessary for integrating technology into teaching processes [51]. The authors defined competencies of teachers as lying both within and at the intersection of the content, pedagogy, and technology elements of the learning environment. For instance, Mishra and Koehler [51] identified technological content knowledge, a competence domain that refers to knowledge about the interaction between content being taught, how the use of technology can facilitate or change content representation, and how the nature of learning specific content changes when technology is integrated into the process. Technological pedagogical knowledge refers to the application of technologies in teaching and learning irrespective of a specific subject area (content knowledge). It includes knowledge of existing tools suitable for supporting learning processes, the ability to choose tools for a specific task or student group, and having a pedagogical strategy for using technological applications [51]. Further development of the model introduced contextual knowledge containing awareness of available technologies and knowledge of the policies of the system in which the teacher operates (eg, school, city, or country) [48]. Adding this aspect to the TPACK model defines another area of knowledge domain teachers need in their professional role and, therefore, suggests that this knowledge domain has to be developed. The TPACK model was selected because it offers a theoretically grounded framework for understanding how technological competencies can be integrated with professional competencies. Although originally developed in educational contexts, its emphasis on the interaction between technological knowledge and professional expertise provides a valuable conceptual foundation for the development of psychotherapeutic competencies in BCP.

#### UTAUT Model

The UTAUT is one of the most frequently used frameworks that have identified factors that promote technology acceptance and predict both behavioral intention to use technology and actual technology use [49]. The following factors were defined in this framework: performance expectancy, effort expectancy, social influence, and facilitating conditions. Performance expectancy refers to the degree to which a user believes the used digital technology will help them succeed in the performance. Effort expectancy is the degree of ease associated with using technology. Social influence refers to the beliefs that important others have about the used technology, whether they think it should be used. Facilitating conditions include beliefs of the user on whether they have the organizational support or technical infrastructure to use the technology in question. It has been shown that the extended UTAUT model, with added barriers, advantages, and knowledge about electronic mental health, explained 74% of the variance in the acceptance of electronic mental health services among psychotherapists in clinical training in Germany and Switzerland [52]. The UTAUT was chosen because it represents most of the frequently used frameworks and because adapted versions specific to BCP already exist [53].

#### Taxonomy of Educational Objectives

In 1956, Bloom [54] introduced the influential Taxonomy of Educational Objectives. This taxonomy was revised in 2002 by Krathwohl [55], who proposed a 2D framework in which the knowledge dimension constitutes the vertical axis and the cognitive process dimension the horizontal axis. The cognitive process categories were partly renamed and are now referred to as remember, understand, apply, analyze, evaluate, and create. These categories are hierarchically ordered but are intended to be applied flexibly rather than as a strictly linear sequence. The knowledge dimension comprises factual, conceptual, procedural, and metacognitive knowledge [55]. The Taxonomy of Educational Objectives was selected because it provides a well-established framework for classifying educational goals, learning objectives, and competence standards [55]. It offers an approach for translating psychotherapeutic competencies into concrete learning outcomes and supports the development of education and training in BCP.

#### Competency Cube

Rodolfa et al [56] have developed a conceptual framework of competence development for professionals in psychology. Their model is presented in the form of a cube with the following dimensions: *Foundational Competency* domains, *Functional Competency* domains, and stages of professional development. *Foundational Competency domains* represent key competence areas of professional psychologists and include Reflective Practice and Self-Assessment; Scientific Knowledge and Methods; Relationships; Ethical and Legal Standards and Policy Issues; Individual and Cultural Diversity; and Interdisciplinary Systems. *Functional Competency domains* include specific services that psychologists provide in their professional role: Assessment and Diagnosis and Case Conceptualization; Intervention; Consultation; Research and Evaluation; Supervision and Teaching; and Management and Administration. Stages of Professional Development refer to different stages of professional psychological education. This model was selected because it is the most influential model of competence development in psychotherapy [57].

#### Telepsychology Practice Model

McCord et al [15] have proposed a consolidated model for telepsychology practice based on an analysis of existing telepsychology guidelines. The model is organized as a cube and includes 3 dimensions: telehealth practice domains, delivery modality, and setting. Telehealth practice domains contain multifaceted professional competencies necessary for the daily professional conduct of psychotherapists. Delivery modality refers to the digital technology or tool used for psychotherapeutic practice. Setting encompasses all possible organizations or institutions where psychologists conduct their practice. This model was selected because it represents one of the most comprehensive competence frameworks specifically developed for telepsychology practice. By addressing psychological competencies across different service delivery modalities and treatment settings, it provides a valuable foundation for understanding how psychotherapeutic competencies may vary according to the mode of delivery.

### Description of the First Development Process

As emphasized before, drawing on these models offers a unique perspective on the issue of competence development. The models that serve as the conceptual foundation of the framework proposed in this paper are highly relevant within their own field of psychology and, therefore, help to gain insight into the necessary components that are to be considered in BCP as well.

The TPACK model [48,51] is a comprehensive model that is intuitively understandable. Therefore, it was used as a baseline to develop a new framework for psychotherapeutic competencies in BCP. We proposed that the conceptualization of the competence of psychotherapists in BCP should be considered at the intersection of psychotherapy and technological (digital) knowledge. Contextual knowledge included in the TPACK model is another important part of the digital competencies of psychotherapists: it is essential that psychotherapists have up-to-date knowledge of current technological solutions in their professional domain and are aware of changing policies in this regard. In the development of our framework, we have used telehealth practice domains and delivery modalities that McCord et al [15] have identified. Moreover, the initial competence domains were derived from the foundational competence domains [56]. Furthermore, the well-established taxonomy of educational objectives [54,55] was incorporated in the initially developed framework to set a baseline for further training development. Grounded in these models, we developed a theory-driven framework including aspects of psychotherapeutic knowledge, technological knowledge, and the overlapping area of psychotherapeutic technological knowledge (PTK). Furthermore, functional and foundational competence domains, as described in the cube model of competencies [56], were included. Therefore, suggestions for the functional and foundational competence domains would be needed specifically for psychotherapeutic approaches in BCP were made. These were integrated into PTK and also brought together with the revised learning objectives by Bloom [54]. This preceding model was presented at the expert workshop. Multimedia Appendix 1 provides a visual overview of the preliminary framework presented during the expert workshop and illustrates how the selected models informed its conceptual structure.

### Expert Workshop

An expert workshop with 5 experts in digital interventions in psychotherapy assessing the proposed model was conducted in September 2023. Experts were identified through purposive sampling by approaching members of relevant professional networks and institutions specializing in digital psychotherapy and BCP, including the eHealth Interest Group of the German Psychological Society (GPS; in German: DGPs), speakers at professional events on digital psychotherapy, and researchers affiliated with relevant academic departments. The final expert panel consisted of 2 professors with established expertise in the field and 3 researchers who had relevant publication records and project experience in BCP. All experts were licensed cognitive-behavioral psychotherapists. None of the experts were part of the author team of this paper. The workshop was conducted over the course of 1 day. It commenced with a presentation of the preliminary version of the framework, outlining its conceptual foundations and structure. Subsequently, a series of structured discussion rounds was conducted in which participants critically examined the preliminary framework and its components. As part of these discussions, a SWOT (strengths, weaknesses, opportunities, threats) analysis was used as a structured method to critically examine this framework. The SWOT analysis is a well-established strategic planning method and one of the most widely used tools for evaluating and developing concepts, processes, and organizational strategies [58]. Due to its structured and flexible nature, it has been applied across a wide range of fields, including education, technology implementation, and health care [59]. Therefore, the method was considered suitable for critically evaluating the preliminary framework and identifying areas for further refinement. Additionally, participants formulated suggestions for improving both the framework itself and its visualization. The insights and recommendations generated across the discussion rounds informed iterative refinements, ultimately leading to the adaptation and further development of the model. To ensure systematic documentation of the workshop, 2 members of the research team (MNW, MT) independently documented the discussions, comments, and recommendations provided by the experts. Furthermore, all materials generated during the workshop, including flipcharts and written documents, were retained. After the workshop, the documented expert feedback and workshop materials were jointly reviewed by the research team and used to identify key recommendations and areas for improvement. These findings informed the subsequent refinement of the framework.

### Systematic Review

After the expert workshop, the integration of findings from a conducted systematic review [43] represented a planned and distinct step in the framework development process. Subsequently, findings from a systematic review on psychotherapeutic competencies in BCP were incorporated to complement the theoretical foundations with an empirical synthesis of the existing literature. The systematic review aimed to identify and synthesize the knowledge, skills, and attitudes required for the implementation of BCP and included 35 studies examining psychotherapeutic competencies in BCP. The review identified 6 thematically overarching Competence Domains [43]. Within the present study, the findings of the systematic review provided an empirical basis for identifying psychotherapeutic competence domains in BCP that are consistently discussed across the existing literature.

### Ethical Considerations

The present study involved the development of a conceptual framework and did not include patients, clinical data, or intervention-related human subjects research. The only direct involvement of individuals consisted of an expert workshop with licensed psychotherapists and researchers with expertise in digital psychotherapy and BCP. The workshop was conducted to obtain expert feedback on a preliminary version of the framework and to support its further refinement. No formal ethics board approval was obtained for the expert workshop, as it did not involve patient data, vulnerable populations, clinical intervention, or the collection of sensitive personal information. Experts were informed about the purpose of the workshop and participated voluntarily. None of the participating experts were members of the author team. Workshop discussions were documented for the purpose of framework development, and results are reported only in synthesized form, without identifying individual participants. Participants of the expert workshop received an expense allowance of €500 (€1=US $1.06 as of September 29, 2023). Travel expenses for train tickets were reimbursed separately, and catering was provided during the workshop day.

## Results

### Results of the Expert Workshop

The expert workshop yielded several key insights that informed the further development of the framework. First, participants emphasized that adopting an educational-psychological perspective within psychotherapy is both valuable and necessary, particularly for structuring training and competence development. Second, they highlighted the importance of differentiating psychotherapeutic practice according to its various Modalities in order to adequately capture the diversity of competencies required in face-to-face, digital, and blended formats. Third, the developed preliminary framework was perceived as offering substantial added value, particularly for educational and training purposes. Furthermore, participants underscored the necessity of defining the term “competence” with greater conceptual precision than the term “knowledge.” Competence, encompassing not only knowledge but also attitudes and skills, facilitates a more comprehensive understanding. Moreover, the experts endorsed the selection of specific domains from the Competency Cube [56] and considered the decision to exclude other domains proposed by Rodolfa et al [56] to be appropriate. Subsequent to the receipt of this feedback, the preliminary model was subjected to refinement and adaptation. Additionally, participants noted that the preliminary framework lacked conceptual clarity, particularly due to its visual presentation based on the original PTK structure. The combination of several Competence Domains within a single tabular illustration was perceived as potentially misleading, giving the impression of 2 parallel frameworks rather than a coherent, unified framework. This feedback highlighted the need for clearer structural logic and a more transparent representation of how the different components relate to one another. Furthermore, the educational objectives [55] were perceived as confusing and overly complex within the framework, and it was suggested that a clear decision be made regarding how the framework should be visualized.

Based on the feedback from the expert workshop, the preliminary framework was subsequently refined. The revised framework clearly distinguishes between *Modalities*, C*ompetence Facets*, and overarching *Competence Domains*, thereby providing a more systematic, internally consistent, and easily interpretable structure. The distinction between delivering modalities and practice domains was furthermore inspired by the cube model by McCord et al [15]. Moreover, we used the term competencies instead of knowledge, as highly recommended during the expert workshop and in line with the existing understanding of the term [22-27]. Furthermore, the educational objectives [55] were excluded from the framework to make it more understandable.

### Revision With the Results of the Systematic Review

Several elements of the earlier version of the framework were revised during this process. First, the previous label “Psychotherapeutic Competence Facets” was replaced with “Competence Domains” to enhance conceptual clarity and consistency within the framework. In addition, the Competence Domains identified through the systematic review were integrated into the framework. These domains include Therapeutic Relationship; Creation of Setting and Infrastructure; Technological Competence; Transfer of Communication and Methods; Self-Regulation, Self-Confidence, and Self-Care; and Data Security, Legal Issues, Ethics, and Safety [43].

### The Final Framework

#### Integration of Competence Domains

The domains from the systematic review [43] show substantial conceptual overlap with the competence areas outlined in the Competency Cube [56], further supporting their relevance and robustness (for comparison, Multimedia Appendix 2). There is 1 domain identified in the systematic review that is not included in the Competency Cube [56] called Technological Aspects. On the other hand, the domains Individual and Cultural Diversity and Scientific Knowledge and Methods are included in the Competency Cube [56] but were not identified as distinct domains in the systematic review [43]. The final framework integrates all of these domains and therefore comprises a total of 8 competence domains.

Importantly, the Technological Competence domain that emerged from the systematic review was renamed Technological Aspects to create greater terminological consistency with the other Competence Domains and to avoid implying a different structural level within the framework. Moreover, we decided to name the domains in line with the results of the systematic review, as a literature-based consensus is included here. For the 2 domains not mentioned in the systematic review, we used the already existing names Individual and Cultural Diversity and Scientific Knowledge and Methods.

#### Conceptual Description of the Framework

The framework encompasses various Modalities of psychotherapeutic delivery, including, for example, videoconferencing, apps, web-based modules, and face-to-face interactions. Modalities are defined as different forms of psychotherapeutic interaction within BCP. Furthermore, the Modalities within the framework can be expanded and therefore offer the possibility of flexible adaptation depending on future developments. Modalities vary in their function (eg, AI-based tools), level of integration into treatment (eg, apps to help implement content into daily life between sessions), and extent of their therapeutic role (unguided vs guided digital interventions). Across all *Modalities*, the 3 *Competence Facets,* defined as including knowledge, skills, and attitudes, represent overarching dimensions of professional competence that apply to each *Modality* and *Competence Domai*n. Thus, the framework implies that specific competence is needed to successfully integrate *Modalities* within BCP. Even unguided digital interventions will need specific knowledge by the therapists to integrate it into an individualized therapeutic plan. Still, we do not rule out overlapping competence factors (eg, a positive attitude concerning technological development in general, easily adapting a Therapeutic Relationship between different Modalities) that help therapists to easily adapt to new developments. Furthermore, there is no hierarchical order intended between Modalities and Competence Facets. The Competence Domains are defined as encompassing thematically related competencies that are relevant for BCP. These Competence Domains are described in the following.

#### Description of Domains

The Therapeutic Relationship domain encompasses the ability to establish an alliance between therapist and patient in BCP. This involves adapting therapeutic competence to digital contexts and dealing with challenges such as reduced nonverbal cues, while also making the most of opportunities for continuity and intimacy through mediated contact [20,60].

The domain Data Security, Legal Issues, Ethics, and Safety covers competencies relating to patient data security and safety, adherence to legal and ethical standards, risk management, and the handling of emergencies and crises in digital and blended settings [11,18,61-63].

The domain Transfer of Communication and Methods comprises competencies that include adapting communication as well as therapeutic methods and interventions to fit BCP, considering patient characteristics, and the appropriateness of interventions for online or face-to-face delivery [18,64].

Self-Regulation, Self-Confidence, and Self-Care describe competencies that include therapists’ ability to manage workload, cope with stress, and maintain self-awareness in BCP [61,65].

The domain Creation of Setting and Infrastructure includes the competence to establish a structured therapeutic environment that ensures privacy, boundaries, and stability while accommodating the flexibility and accessibility of BCP modalities [18,19,66].

Technological Aspects encompass the competence to use and manage digital tools in order to guarantee stable and secure communication. Furthermore, technical proficiency, an understanding of digital tools, the capacity to resolve technical issues, and the ability to integrate technology into therapeutic processes are mentioned here [18,43,61].

The competence domain of Individual and Cultural Diversity refers to awareness and sensitivity when working with diverse individuals, groups, and communities that represent a wide range of cultural and personal backgrounds and characteristics [56].

The domain Scientific Knowledge and Methods encompasses the ability to understand and apply research and research methodologies, as well as respect for scientifically derived knowledge. This includes familiarity with techniques of data collection and analysis, knowledge of the biological bases of behavior, cognitive-affective processes, and human development across the lifespan [56]. This domain is highly relevant, especially in a time of fast development of Modalities.

#### Description of Competence Clusters

Furthermore, consistent with the approach used in the systematic review [43], the *Competence Domains* were clustered into 3 overarching thematic areas. We expand this here by integrating the 2 additional competence domains drawn from Rodolfa et al [56]. The *Foundational Cluster* contains the domains Data Security, Legal Issues, Ethics, and Safety, and Technological Aspects, as well as Creation of Setting and Infrastructure. This cluster provides the structural, technical, and legal conditions necessary for BCP and is strongly supported by existing competence frameworks for digital competencies [21,25,45]. Therefore, this domain describes general psychotherapeutic competencies that are highly important for the context of BCP. The *Psychotherapeutic Process Cluster* encompasses the Therapeutic Relationship domain as well as the Transfer of Communication and Methods. This cluster is relevant to the core therapeutic process, capturing the ways in which psychotherapists modify relational, communicative, and methodological practices for BCP. The competence domain Transfer of Communication and Methods is specific to BCP contexts [43]. Even if the Therapeutic Relationship is relevant in psychotherapy generally, it differs in BCP [43,61]. The *Reflexive Cluster* incorporates the domain of Self-Regulation, Self-Confidence, and Self-Care. We decided to include the domains *Individual and* Cultural Diversity and Scientific Knowledge *and Methods* taken from the cube model [56] within this cluster. Accordingly, the *Reflexive Cluster* does not only capture those competence domains that relate to ongoing self-reflection and one’s professional self-awareness. It further contains the critical evaluation of one’s therapeutic practice, reflecting on the effect of diversity and culture within the practice of BCP, as well as the willingness and insight into the necessity of constant training and adaptation to new insights brought by new scientific knowledge. This cluster extends previous existing competence frameworks in digital contexts [43]. Although they already exist in general psychotherapeutic competencies [56,57,67], they seem highly relevant in the rapidly developing field of BCP [13,41].

**Figure 1.**
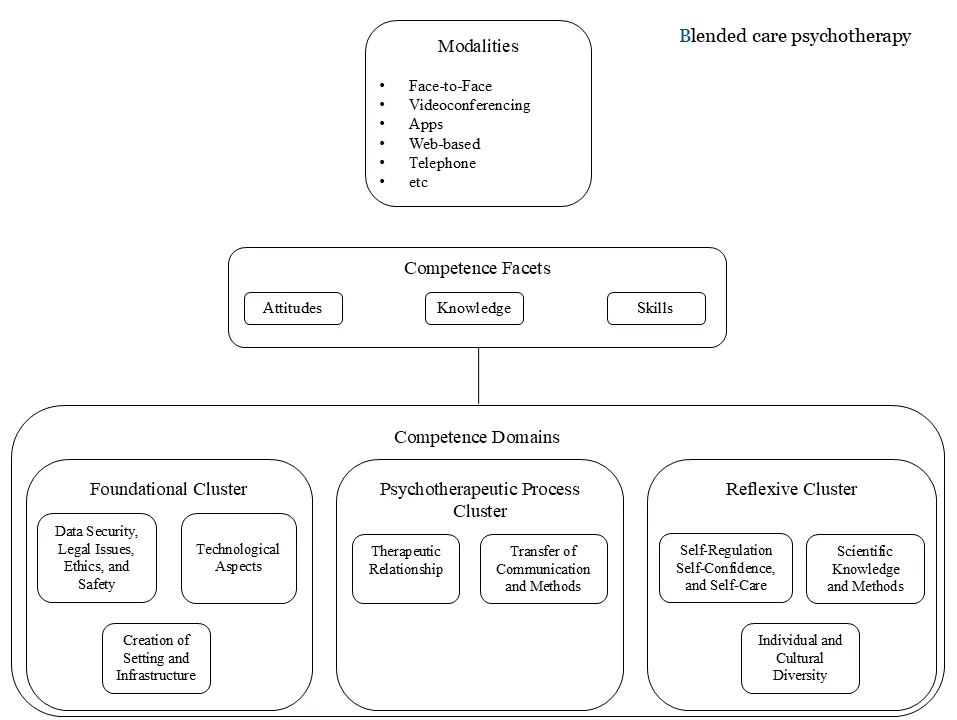
Framework of psychotherapeutic competencies in blended care psychotherapy.

To facilitate understanding of the framework (Figure 1), the following example illustrates how its components interact in practice. For example, the use of videoconferencing (*Modality*) is shaped by the interplay of knowledge, skills, and attitudes (*Competence Facets*) across the 8 *Competence Domains* outlined in the framework. Within the *Competence Domain* of Technological Aspects, for example, relevant knowledge includes understanding available videoconferencing tools and their functionalities (eg, platform features). Correspondingly, specific skills are required, such as the ability to operate the selected platform and to troubleshoot basic technical issues. Attitudes also play a crucial role, as they influence whether, when, and how frequently psychotherapists choose to use videoconferencing in their practice and which tool (Technological Aspects) they might choose. Furthermore, these attitudes may include beliefs about the effectiveness of video-based interventions, ethical considerations related to digital communication, and perceptions of the feasibility of establishing and maintaining a therapeutic relationship via videoconferencing.

## Discussion

### Development and Validation of the BCP Competence Framework

The present paper develops a competence framework for BCP by integrating established models from educational and clinical psychology, formative feedback from an expert workshop, and evidence synthesized through a systematic review. The framework should, therefore, be understood as theoretically grounded and evidence-informed, but not yet externally or empirically validated.

### Theoretical Contributions of the Framework

First, existing models of technological competence (TPACK) [48,51], educational perspectives (learning objectives) [54,55], psychotherapeutic competence (Competency Cube) [56], and competencies for technological practice [15] have been used as a baseline to inform the development of our framework. Moreover, the UTAUT [49] informed the development of the framework, as reflected in the emphasis on the competence facet of attitudes. By integrating findings from an expert workshop in combination with a systematic review, the framework contributes to the theoretical consolidation of what constitutes professional competence in BCP. In more detail, the present framework extends these foundational theories and frameworks by applying them to address competencies of psychotherapists working in the digital context. Our framework shows that specific competencies are needed beyond those related to the use of technology.

Second, the framework provides conceptual clarity in an area where terminological inconsistencies are common [68]. By articulating Competence Domains specific to BCP and aligning them with established competence structures, the model contributes to the theoretical refinement of competence-based approaches in psychotherapy. It underscores the importance of understanding competencies as incorporating knowledge, skills, and attitudes that are needed across digital and analog Modalities [26]. Finally, this work highlights the theoretical need for a dynamic understanding of competence, which is essential because digital technologies and therapeutic tools change rapidly, and the competence landscape in psychotherapy cannot be regarded as static [69].

The framework, therefore, supports a more adaptive and context-sensitive conceptualization of psychotherapeutic competence. This approach can accommodate technological innovation and evolving clinical formats that can be integrated into the modality component of the framework. This theoretical perspective may inform future models of professional development and learning within psychotherapy, in line with the perspective of Rief [70], which postulates that competence profiles, the optimization of training and education as well as the reality in science and practice inform each other.

With regard to the competence domains, it should be emphasized that the inclusion of the 2 domains derived from the Competency Cube [56] is consistent with recent literature. For example, the domain *Research and Evaluation in Digital Psychological Practice*, identified in a recent review of digital psychological interventions [47], highlights the importance of scientific knowledge and the ability to apply research skills in digital contexts. This shows clear parallels to the domain *Scientific Knowledge and Methods* [56] and supports the relevance of this competence within BCP. Similarly, the domain *Access and Equity in Digital Practice*, also identified by Kule et al [47], emphasizes the importance of reaching underserved populations and ensuring equitable and inclusive digital care. This demonstrates conceptual overlap with the domain Individual and Cultural Diversity [56], further reinforcing the need to integrate this competence into BCP frameworks. It should be noted that these 2 domains did not emerge as distinct domains in the systematic review [43]. It is possible that these 2 domains are not yet represented in empirical research on psychotherapeutic practice. Moreover, it should be noted that all Competence Domains identified in the scoping review on digital competencies of psychotherapists [47] are also reflected in our systematic review on psychotherapeutic competencies in BCP [43], with the exception of the domain Self-Regulation, Self-Confidence, and Self-Care.

Importantly, the resulting framework is intended to be applicable across psychotherapeutic orientations, as the identified Competence Domains reflect overarching psychotherapeutic competencies relevant to BCP more generally.

### Practical Implications

Beyond its theoretical contributions, the competence framework also holds significant practical implications for professional training, clinical practice, and organizational implementation of BCP [13,15]. The 8 competence domains provide clear targets for competence development, enabling educators to design learning activities, assessments, and supervision practices that systematically build students’ and practitioners’ readiness to work in BCP. At an organizational level, the framework may guide the development and implementation of organizational training programs for therapists and psychotherapists in training, support the establishment of quality standards for the use of digital tools in psychotherapy, and inform service protocols regarding data protection, ethical considerations, and the technological infrastructure required for BCP. Furthermore, the framework could support the development of accreditation standards or best-practice guidelines for digital mental health, ensuring that therapists’ competencies are systematically defined and evaluated.

### Limitations

Some limitations of this framework development should be acknowledged. Although the expert workshop provided valuable insights for refining the competence framework, the number of participants may limit the breadth of perspectives represented. As with most expert-based approaches, the findings are influenced by the specific backgrounds, clinical experiences, and theoretical orientations of the individuals involved [71]. However, despite the fact that all participants were cognitive-behavioral therapists, they possessed divergent levels of practical and scientific experience and had been involved in a variety of projects and perspectives in the field of BCP. Additionally, the expert workshop was only 1 step in the development process. The specificity of the workshop can therefore be considered a strength, in contrast to the expanded literature search of the systematic review, including a qualitative approach within a multimethod development process.

Furthermore, the framework was developed within a specific technological and regulatory landscape. Digital tools and data protection requirements evolve rapidly, and competence needs may shift accordingly [69]. This raises questions about the long-term applicability of the framework and underscores the need for regular updates as digital mental health practice continues to develop. Notwithstanding the aforementioned limitations, the provided model offers a solid foundation upon which to base future changes, including developments in the field of AI that change not only the landscape of digital tools but also multiple Competence Domains of psychotherapists (eg, Technological Aspects and Scientific Knowledge and Methods).

Another limitation concerns the perspectives included in the framework development process. The framework was developed to reflect psychotherapists’ perspectives. However, psychotherapeutic competencies are inherently relational and may also be shaped by patients’ needs and experiences. This is supported by previous research into patients’ perspectives on the implementation of BCP, which identified therapist behavior as a crucial motivational factor [5]. Therefore, future research should incorporate patients’ perspectives to examine whether the competencies identified in the present framework correspond to patients’ needs and experiences.

Although the expert workshop provided important formative feedback on the preliminary framework and informed its subsequent refinement, the final version has not yet been externally or empirically validated. Future research is therefore needed to examine the validity and distinctiveness of the proposed domains, investigate how these competencies manifest in practice, determine how they can be reliably assessed, and evaluate whether they predict treatment quality or outcomes in BCP. This, of course, includes the operationalization of certain aspects of the model such as attitudes. However, the UTAUT might be an easy solution, as it is one of the most widely applied models in technology acceptance research and identifies key factors that predict both behavioral intention and actual technology use [49].

### Future Research Directions

The development of this competence framework highlights several important avenues for future research. First, empirical validation of the identified competence domains represents a critical next step. Quantitative studies, such as factor-analytic approaches, expert ratings, or competence assessments, are needed to examine the structure, distinctiveness, and coherence of the domains [72,73]. Validation efforts should also investigate whether the proposed competencies predict relevant clinical outcomes, such as treatment adherence, the therapeutic alliance in digital formats, or patient improvement in BCP.

Second, future research should focus on operationalizing the Competence Domains into measurable indicators that can inform training, supervision, and professional development. Developing standardized assessment tools or behavioral markers would allow educators and institutions to evaluate therapists’ competence levels and monitor competence acquisition over time. For practitioners, this could offer guidance for self-assessment and professional development. Therapists could use self-evaluation scales to assess their strengths and developmental needs in psychotherapy in the field of digital elements as well as face-to-face elements. This encourages psychotherapists to reflect on their practice and helps them navigate the demands of an evolving field of psychotherapy. Finally, the perspective of all stakeholders, including patients, should be evaluated in future research [68].

### Conclusions

This study presents an evidence-informed and expert-supported framework of psychotherapeutic competencies for BCP. The framework offers a conceptually robust and practically relevant structure for understanding the knowledge, skills, and attitudes required for competent psychotherapeutic work in digitally supported contexts. The refinement of the framework, including the differentiation of Modalities, Competence Domains, and Competence Aspects, enhances its clarity and usability for both educational and clinical purposes. The resulting framework contributes to theoretical conceptualizations of competence in digital psychotherapy and provides a foundation for developing targeted training programs that prepare psychotherapists, trainees, and students for the demands of blended care practice. Given the rapid expansion of digital technologies in mental health care, the delineation of competencies specific to BCP represents a timely and necessary step toward ensuring high-quality treatment delivery. Future research should aim to empirically validate the proposed Competence Domains, investigate their predictive value for clinical processes and outcomes, and develop reliable assessment tools grounded in this framework. Such efforts will be essential for establishing competence-based training standards and fostering the safe and effective integration of BCP Modalities into psychotherapeutic practice.

## Supplementary material

10.2196/100125 Multimedia Appendix 1 Insight from the expert workshop.

10.2196/100125 Multimedia Appendix 2 Comparison of the Competence Domains.

## Abbreviations

BCP: blended care psychotherapy
GPS: German Psychological Society
PTK: psychotherapeutic technological knowledge
SWOT: strengths, weaknesses, opportunities, and threats
TPACK: Technological Pedagogical Content Knowledge
UTAUT: unified theory of acceptance and use of technology
