# Supplementary material for: Psychotherapeutic Competencies in Blended Care Psychotherapy: Development of a Competence Framework
Source: JMIR Ment Health. 2026 Sep 16;13:e100125. doi: 10.2196/100125 (PMC13581150; doi:10.2196/100125)
Supplement: Supplementary file 1 [file mental-v13-e100125-s001.docx]

**Appendix 1**

**Insight from the expert workshop**

**Figure A1.1**

*First illustration of the framework.*

*Note. PTK= Psychotherapeutic Technological Knowledge. Building on the TPACK model*^1^*, this representation was adapted to the BCP context by conceptualizing psychotherapeutic competences as emerging from the integration of psychotherapeutic and technological knowledge.*

**Figure 1.2**

*Selection of Competence Domains from the Competence Cube*^2^

*Note. The grey colored competences are the selected competence domains that were shown and approved in the expert workshop.*

**Figure 1.3**

*List-based illustration of the Competence Framework.*

*Note. TK= Technological Knowledge, PTK= Psychotherapeutic Technological Knowledge, R=Remember, U=Understand, A= Apply, An= Analyze, Ev= Evaluate, C=Create. The revised Taxonomy of Educational Objectives*^3^ *was incorporated to support the translation of BCP competences into structured learning objectives for future training development.*

**References**

1.	Mishra P, Koehler MJ. Technological Pedagogical Content Knowledge: A Framework for Teacher Knowledge. 2006.

2.	Rodolfa E, Bent R, Eisman E, Nelson P, Rehm L, Ritchie P. A Cube Model for Competency Development: Implications for Psychology Educators and Regulators. Prof Psychol Res Pract. 2005 Aug;36(4):347–54. doi:10.1037/0735-7028.36.4.347

3.	Krathwohl DR. A Revision of Bloom’s Taxonomy: An Overview. Theory Pract. 2002 Nov 1;41(4):212–8. doi:10.1207/s15430421tip4104_2
