# Supplementary material for: Psychotherapeutic Competencies in Blended Care Psychotherapy: Development of a Competence Framework
Source: JMIR Ment Health. 2026 Sep 16;13:e100125. doi: 10.2196/100125 (PMC13581150; doi:10.2196/100125)
Supplement: Supplementary file 2 [file mental-v13-e100125-s002.docx]

**Appendix 2**

**Comparison of the Competence Domains**

In the following table, the comparison between the specific competence domains derived from the Competency Cube^1^ reviewed and refined in an expert workshop and the systematic review^2^ is presented. This comparison revealed substantial overlap between the two sources. However, two domains included in the Competency Cube *Individual–Cultural-Diversity* and *Scientific-Knowledge-Methods* were not identified in the systematic review. Conversely, one domain emerging from the systematic review, *Technological Competence,* was not represented in the Competency Cube. A detailed comparison of these domains can be seen in Table 1.

**Table 2.1**

*Comparison of Competence Domains: Competency Cube ¹ and Systematic Review ²*

| Competency Cube  Domain | Competence | Systematic Review  Domain | Competence |
| --- | --- | --- | --- |
| Relationships | Capacity to relate effectively and meaningfully with individuals, groups, and/or communities. | Therapeutic Relationship | Competence to create an alliance between therapist and patient characterized by empathy. It involves adapting therapeutic competences to digital contexts, addressing challenges such as reduced non-verbal cues while leveraging opportunities for continuity and intimacy through mediated contact. |
| Ethical–legal standards–policy | Application of ethical concepts and awareness of legal issues regarding professional activities with individuals, groups, and organizations. Advocating for the profession. | Data Security, Legal Issues, Ethics and Safety | Competences related to patient data security, ensuring safety, adhering to legal and ethical standards, managing risk, and effectively addressing emergency or crisis situations within digital and blended care contexts. |
| Assessment–diagnosis–case conceptualization  AND  Intervention | Assessment and diagnosis of problems and issues associated with individuals, groups, and/or organizations.  Interventions designed to alleviate suffering and to promote health and well-being of individuals, groups, and/or organizations. Understanding of empirically supported treatments. | Transfer of Communication and Methods | Competences that include adapting communication as well as therapeutic methods and interventions to fit BCP, considering patient characteristics, technological limitations, and the appropriateness of interventions for online or face-to-face delivery. |
| Reflective practice self-assessment | Practice conducted within the boundaries of competences, commitment to lifelong learning, engagement with scholarship, critical thinking, and commitment to the development of the profession. | Self-Regulation, Self-Confidence and Self-Care | Competence that includes therapist ability to manage workload, cope with stress, and maintain self-awareness in BCP. |
| Management–administration | Managing the practice of mental health services and/or the administration of health organizations, programs, and agencies. | Creation of Setting and Infrastructure | Competence to establish a structured therapeutic environment that ensures privacy, boundaries, and stability while accommodating the flexibility and accessibility of digital modalities. |
|  |  | Technological Competence | Competence to utilize and manage digital tools in order to guarantee stable and secure communication. This encompasses technical proficiency, an understanding of digital tools, the capacity to resolve technical issues, and the ability to integrate technology into therapeutic processes. |
| Individual–cultural diversity | Awareness and sensitivity in working professionally with diverse individuals, groups, and communities who represent various cultural and personal background and characteristics. |  |  |
| Scientific knowledge–methods | The ability to understand research, research methodology and a respect for scientifically derived knowledge, techniques of data collection and analysis, biological bases of behavior, cognitive-affective bases of behavior, and lifespan human development. |  |  |

**References**

1.	Rodolfa E, Bent R, Eisman E, Nelson P, Rehm L, Ritchie P. A Cube Model for Competency Development: Implications for Psychology Educators and Regulators. Prof Psychol Res Pract. 2005 Aug;36(4):347–54. doi:10.1037/0735-7028.36.4.347

2.	Woide MN, Weiher GM, Zirenko M, Tremmel M, Scherer S, Ochmann MK, et al. Psychotherapeutic competencies for blended care psychotherapy: A systematic review of knowledge, skills, and attitudes. Clin Psychol Sci Pract. 2026 Jun 8. doi:10.1037/cps0000348
